# Sea level rise, surface warming, and the weakened buffering ability of South China Sea to strong typhoons in recent decades

**DOI:** 10.1038/s41598-017-07572-3

**Published:** 2017-08-07

**Authors:** Jingru Sun, Leo Oey, F.-H. Xu, Y.-C. Lin

**Affiliations:** 1 Ministry of Education Key Laboratory for Earth System Modeling, Department of Earth System Science, Tsinghua University, Beijing, 100084 China; 2 Graduate Institute of Hydrological & Oceanic Sciences, National Central University, 300 Zhongda Road, Jhongli City, Taoyuan County, 320 Taiwan; 3 Princeton University, Princeton, 08540 New Jersey USA

## Abstract

Each year, a number of typhoons in the western North Pacific pass through the Luzon Strait into South China Sea (SCS). Although the storms remain above a warm open sea, the majority of them weaken due to atmospheric and oceanic environments unfavorable for typhoon intensification in SCS, which therefore serves as a natural buffer that shields the surrounding coasts from potentially more powerful storms. This study examines how this buffer has changed over inter-decadal and longer time scales. We show that the buffer weakens (i.e. greater potential for more powerful typhoons) in negative Pacific Decadal Oscillation (PDO) years, as well as with sea-level-rise and surface warming, caused primarily by the deepening of the ocean’s 26 °C isotherm *Z*_*26*_. A new Intensity Change Index is proposed to describe the typhoon intensity change as a function of *Z*_*26*_ and other environmental variables. In SCS, the new index accounts for as high as 75% of the total variance of typhoon intensity change.

## Introduction

**Figure 1:**
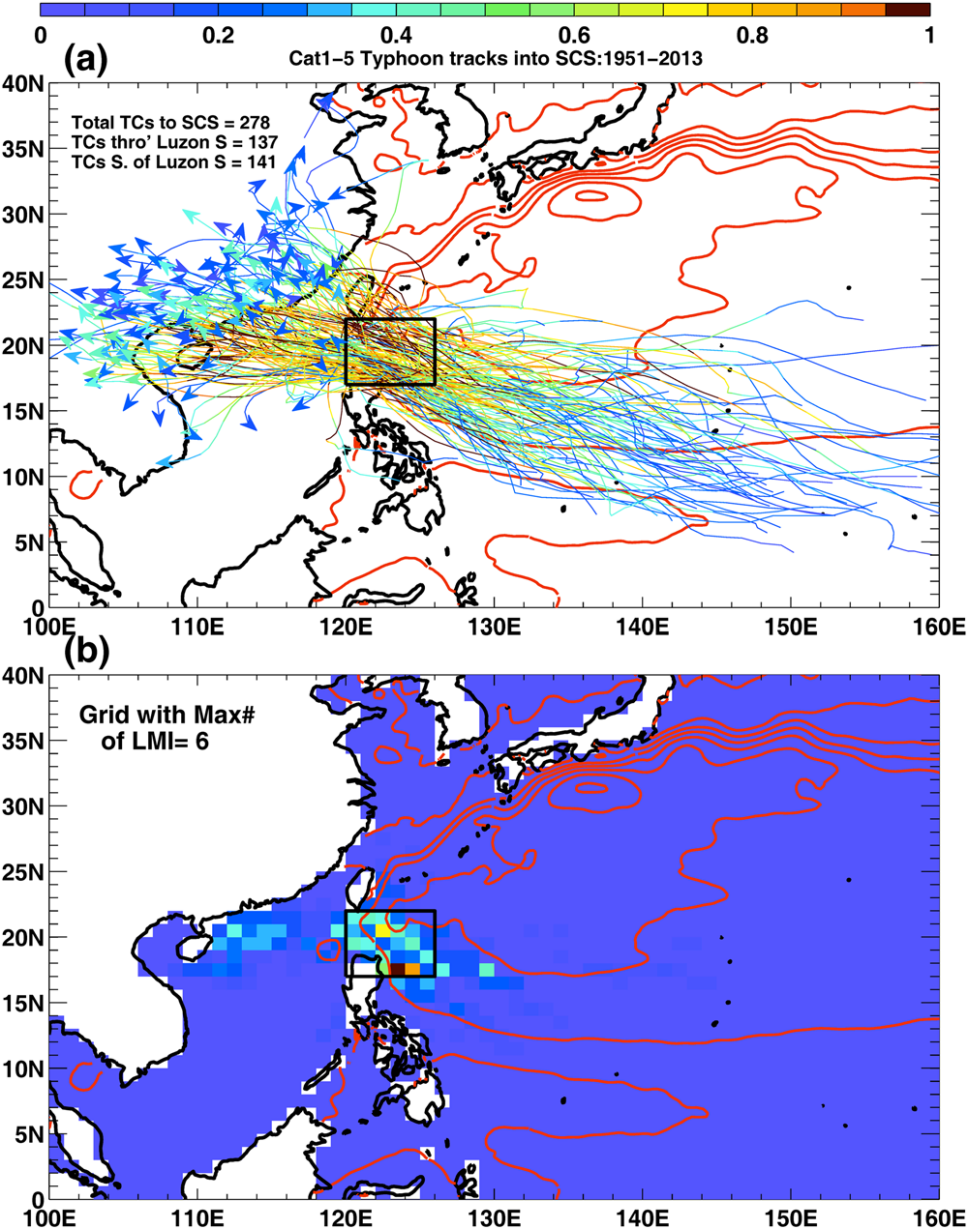
(**a**) Tracks of typhoons from western Pacific into SCS through the Luzon Strait, from 1951–2013, updated from Sun and Oey^1^ using the latest IBTrACS observations. For each track, colors show 6-hourly wind speeds normalized by the maximum wind speed for that track; darkest brown color corresponds to normalized wind speeds >0.95, and indicates where and when the typhoon was near its LMI. Top left shows the total TCs into SCS (278), subdivided into those passing through the Luzon Strait (i.e. the LSTCs, 137), and south of the strait (141, tracks not shown). Red contours show mean absolute dynamic topography MADT (*m*) from AVISO http://www.aviso.oceanobs.com/. (**b**) The number of LMIs per 1^o^ × 1^o^ grid (normalized by the maximum number = 6), according to tracks in (**a**). Tracks within 50 km of the northern (southern) tip of Luzon (Taiwan) were excluded. Maps plotted using MATLAB Version#R2012a (7.14.0.739) 64-bit (glnxa64) (https://www.mathworks.com/support/sysreq/previous_releases.html).

According to the data from the International Best Track Archive for Climate Stewardship (IBTrACS), approximately half of the total number of tropical cyclones (Category 1 and above, hereinafter TCs or typhoons) that enter the South China Sea (SCS) from western North Pacific pass through a narrow gap between Luzon and Taiwan: the Luzon Strait (Fig. 1a)^1^. These Luzon Strait TCs (LSTCs) remain above the ocean throughout their lives, minimally affected by terrain^1^, and nearly all of them make landfall along southern China and Vietnam, causing considerable property losses and human sufferings^2, 3^. The coasts of southern China and Vietnam have in fact been identified to be amongst the world’s most vulnerable to floods from TCs in future projected sea-level rise under climate change scenarios^4, 5^. The majority of LSTCs reached their maximum intensities in the warm waters east of Luzon and in the Kuroshio, and then weakened in the SCS^1^. The fact that the majority of LSTCs weaken after passing through the Luzon Strait suggests the existence of some common environmental factors in the ocean and/or atmosphere which are unfavorable to TC-intensity in SCS, thus making SCS a natural “buffer” which shields southern China and Vietnam from otherwise stronger typhoons from the open western Pacific. This study seeks to describe and understand what factors contribute to the buffer and how the buffer has changed over inter-decadal periods based on observations and reanalysis data. It should be noted that the same environmental factors exist, and our results would apply also to TCs that enter the SCS south (north) of the Luzon Strait; however, the intensities of these TCs may also be strongly affected by the mountainous terrain of Luzon (Taiwan). The LSTCs represent an ideal limiting group of TCs with the maximum potential of reaching their lifetime maximum intensities (LMIs) inside SCS. Besides satisfying our scientific curiosity, knowledge about the behaviors and variability of LSTCs and their dependence on the buffer can help improve storm risk assessments, which are required to formulate plans for long-term adaptation and storm preparedness. We will show that the buffer has weakened since the mid-1980s and early 1990s – coincident with the beginning of rapid rise in sea surface temperature (SST) and sea level^6, 7^.

## Results

Figure 1a (dark brown color along each track) shows the location where the TC is near its LMI, and Fig. 1b the number of LMIs per 1° × 1° grid. The majority of LSTCs (70%) reach their peak intensities in the western Pacific (WP), and weaken as they enter the SCS^1^. The remaining 30% continue to intensify and reach peak intensities inside the SCS, mostly over the warm waters of the northern SCS shelves prior to landfalls (Fig. 1b). The SCS therefore serves as a natural buffer to dampen TCs. What are the dominant oceanic and/or atmospheric environmental factors that control the SCS buffer, and how do they vary over inter-decadal and longer time scales?

### PDO and SCS buffer

**Figure 2:**
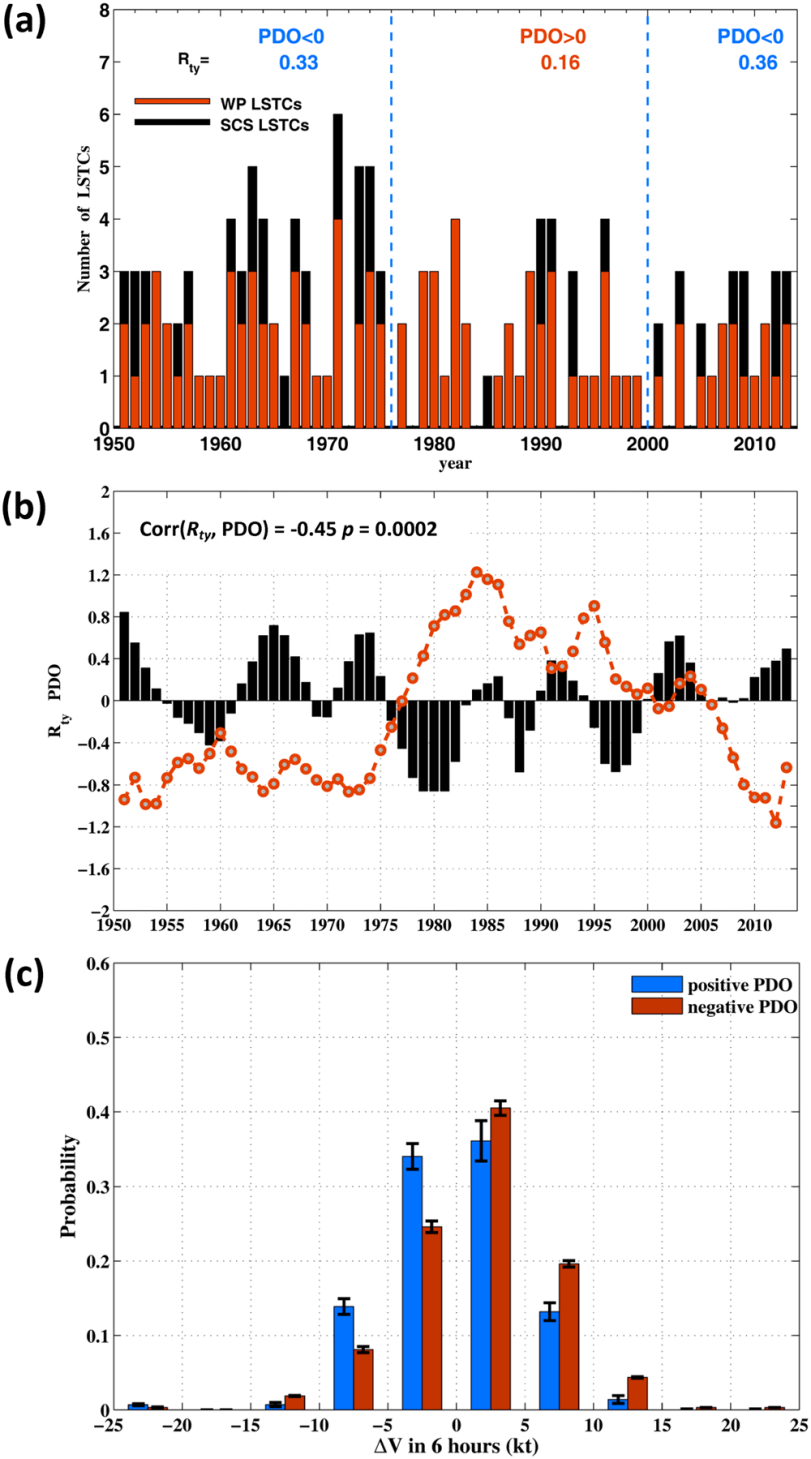
(**a**) Numbers of WP-(red bars) and SCS-(black bars) intensifying LSTC’s based on the IBTrACS data from 1951–2013; top display shows values of *R*_*ty*_ averaged over periods of positive and negative PDOs indicated by vertical dashed lines. (**b**) *R*_*ty*_ anomaly normalized by its standard deviation σ (s.d. = 0.27) (black bars) and the PDO index (red dashed line), 6-year running mean applied to both; a tapered smoothing is applied to end points; top-left display shows the correlation between *R*_*ty*_ and PDO and the corresponding *p*-value. (**c**) Probability distribution of 6-hourly intensity changes (ΔV) of LSTCs in SCS sampled by positive and negative PDOs. Units are knots [1 knot (kt) = 0.51 m s^−1^] per 6 hours. Data are in bins of 5-kt resolution. Error bars show ±σ from the mean probability for that bin.

The Pacific Decadal Oscillation^8^ (PDO) and the El Niño Southern Oscillation^9^ (ENSO) can potentially affect the SCS buffer. TC activity depends on the phases of ENSO and PDO^3, 10–13^. We focus on inter-decadal changes and begin with an examination of how the SCS buffer varies with PDO. As a measure of the buffer’s strength, we define the ratio:

1 $${R}_{ty}{={\rm{N}}}_{{\rm{SCS}}}/({{\rm{N}}}_{{\rm{SCS}}}{+{\rm{N}}}_{{\rm{WP}}}){={\rm{N}}}_{{\rm{SCS}}}/{{\rm{N}}}_{{\rm{TOT}}},$$

where N_SCS_ (N_WP_) is the number of LSTCs per typhoon season from June through November that intensify (i.e. have LMIs) in SCS (WP), and N_TOT_ = N_SCS_ + N_WP_ is the total number of LSTCs per season. The *R*_*ty*_ ≤ 1, and a smaller (larger) ratio indicates a stronger (weaker) buffer when less (more) LSTCs intensify in SCS. For the analysis period from 1951 to 2013, the *R*_*ty*_ is larger (mean = 0.34) during negative PDO (denoted by −PDO) years, and smaller (mean = 0.16) during positive PDO (denoted by + PDO) years (Fig. 2a). The *R*_*ty*_ and PDO appear to be anti-correlated, Corr(*R*_*ty*_, PDO) = −0.45 (Fig. 2b); however, the sample is too short for their connection to be statistically meaningful, and another measure is described below. Nonetheless, it appears that a larger percentage of typhoons would gain strength and reach maximum intensity in SCS during the −PDO than the +PDO years after passing through the Luzon Strait.

A number of studies^3, 14^ have shown that during −PDO (+PDO) years, typhoons tend to form farther west (east) in the tropical North Pacific (see Supporting Information Fig. S1), resulting in more (less) typhoons entering the South China Sea (Fig. S2). Between 1951 and 2013, the average number of LSTCs per season is 2.6 during the −PDO years, and 1.2 during the +PDO years. However, since *R*_*ty*_ is a ratio, a larger supply of LSTCs (i.e. N_TOT_) during −PDO years does not necessarily lead to a higher percentage of typhoons that intensify in SCS, i.e. to a larger *R*_*ty*_. The probability distribution of intensification rates (ΔV) of LSTCs in SCS in fact shows that the mean intensification rates between +PDO and −PDO years are well separated. For a wide range −10 ≤ ΔV ≤ 15 kt per 6 hours, the intensification rates are positive during −PDO and negative during +PDO (Fig. 2c). The result is consistent with the apparent anti-correlation between *R*_*ty*_ and PDO, noted above. Changes in *R*_*ty*_ are therefore more likely caused by changes in the oceanic and atmospheric environments, which will be examined next.

### Environmental conditions

TC internal dynamics play an important role in intensity changes^15^. To study inter-decadal variation of LSTCs, we examine instead how large-scale environmental conditions have evolved, and seek some quantifiable metric(s) to describe the change. Environmental factors known to affect storm intensity are: (i) relative humidity (*RH*)^16–22^, (ii) environmental vertical wind shear **V**_s_ of the horizontal wind^16, 20, 23–28^, (iii) large-scale vorticity (VOR)^16, 24^, (iv) SST^16, 20, 24, 29–32^, (v) maximum potential intensity (MPI)^20, 28, 32^, and (vi) the depth of upper-ocean warm layer, measured for example by the depth *Z*_26_ of the 26 °C isotherm^1, 15, 18, 32, 33^. For a shallow *Z*_26_, typhoon winds can more easily mix and entrain cold subsurface water to the surface, lowering the SST and reducing the storm intensity or even killing it. On the other hand, for thick *Z*_26_, the SST may remain above 26 °C despite the strong wind mixing, allowing the storm to maintain its strength or even to intensify. We calculate TC maximum potential intensity, MPI(SST) (m s^−1^), which depends on SST and the structure of the overlying atmosphere^34, 35^. Price^36^ suggested that, due to mixing by the strong TC wind, a temperature (denoted here by T_mix_) depth-averaged over some depth h_mix_ would more realistically approximate the SST under the TC; the idea has been applied in various TC-genesis and intensity-change studies^37–39^. Here we calculate T_mix_ by assuming a linear temperature profile between the surface and *Z*_*26*_ below the surface, which is then averaged over h_mix_:

2 $${{\rm{T}}}_{{\rm{mix}}}=\text{SST}\,-\,({\rm{SST}}\,-\,26){\text{h}}_{\text{mix}}/(2{Z}_{26}).{H}_{v}(\text{SST}-26),$$

where *H*_*v*_ is the Heaviside function. A value of h_mix_ = 80 m is used^37^. Equation (2) shows that, as the *Z*_*26*_ deepens, T_mix_ increases to approach the temperature of the warmed ocean surface where the SST is warmer than 26 °C, thus modeling the sheltering characteristic of a deep and warm upper-ocean layer. On the other hand, when *Z*_*26*_ decreases, T_mix_ becomes increasingly cooler than SST, thus modeling the cooling due to the more ready entrainment of cold water from beneath the shallower warm surface layer. As SST ≤ 26 °C, T_mix_ is set to SST. The *Z*_*26*_ is calculated using the monthly WOA data plus an anomaly *Z*_*26*_’. The ocean is divided into 2 layers separated by the 26 °C isotherm. Monthly *Z*_26_ and density difference Δρ/ρ_o_ between the 2 layers are calculated using WOA^40, 41^ (Fig. S3); then the sea-level anomaly (SLA = η’)^42, 43^ from AVISO is used to obtain *Z*′_26_ ≈ η′ρ_o_/Δρ. The MPI based on T_mix_ is denoted as MPI(T_mix_).

### Intensity Change Index (*ICI*)

We develop an index to describe the above environmental effects on TC intensity change (see Methods):

3 $$ICI={(\frac{RH}{50})}^{0.8}\,{(\frac{MPI}{70})}^{3.2}\,{(1+0.1\times WSH)}^{-1.6}.$$

The *ICI* is similar in form to the Genesis Potential Index (GPI)^44, 45^. However, while GPI is useful for studying preferred locations of TC genesis as a function of environmental conditions, we show below that *ICI* is a preferred index for studying TC intensity change.

### Composites for +PDO and −PDO phases

**Figure 3:**
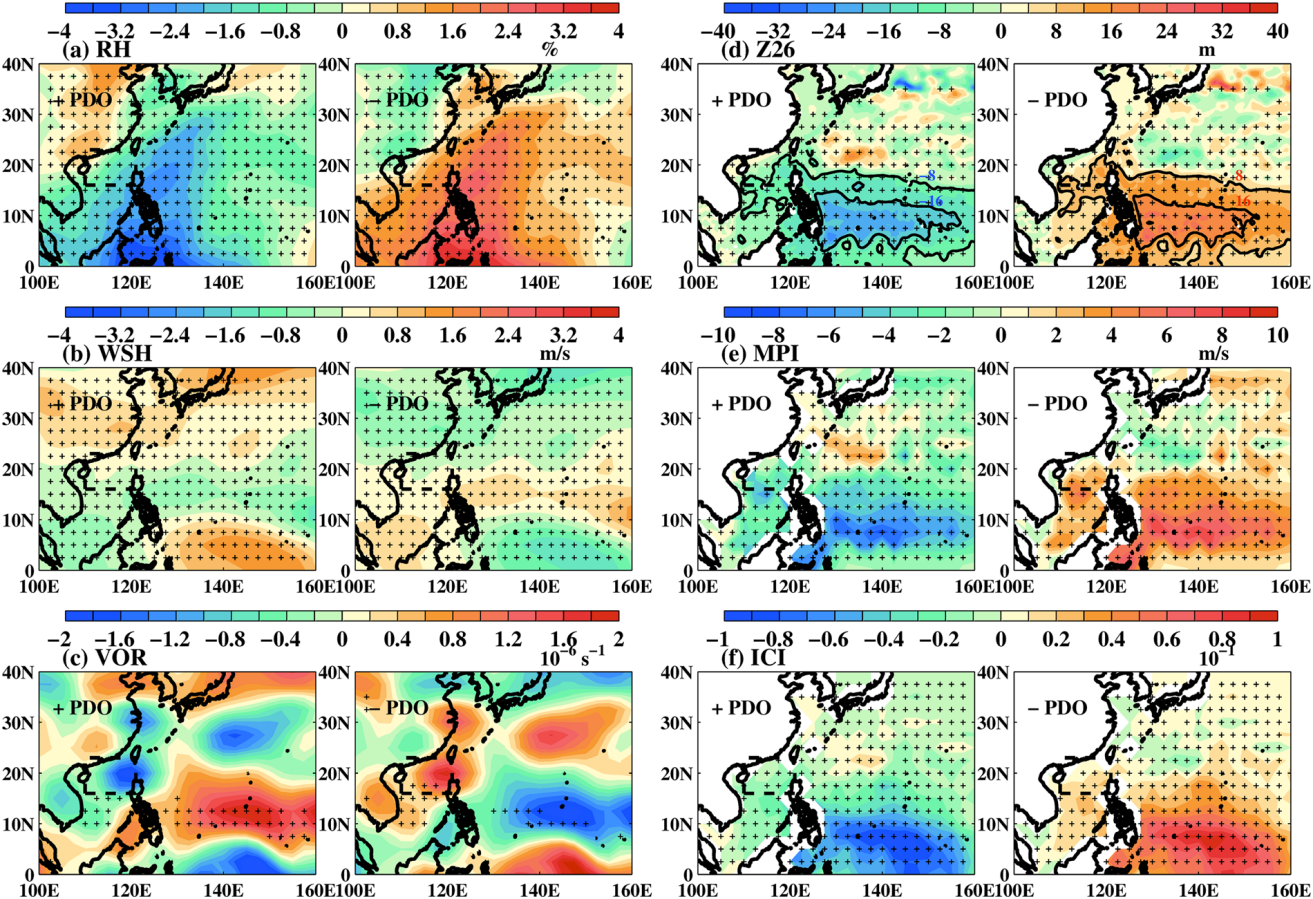
Composites of (**a**) RH, (**b**) WSH, (**c**) VOR, (**d**) *Z*_*26*_, (**e**) MPI and (**f**) *ICI* for +PDO and −PDO from 1992 to 2013. Since +PDO is from 1992~2005, while −PDO is from 2005~2013, each pair of plots approximately describes the decadal change between the two epochs. Stipples indicate regions where the composite mean is significant. Dashed box in northern SCS shows the main area of interest. In each panel abscissa is longitude and ordinate is latitude. Maps plotted using MATLAB Version#R2012a (7.14.0.739) 64-bit (glnxa64) (https://www.mathworks.com/support/sysreq/previous_releases.html).

We composite the above variables according to +PDO and −PDO during the satellite altimetry period from October 1992 to 2013 (Fig. 3). We focus in the northern SCS (15–24N & 110–121E where all LSTCs pass) and use the notation Δ_−+_(α) to denote the difference in the variable α: −PDO composite minus +PDO composite. Since +PDO is from 1992~2005 and −PDO from 2005~2013 (see Fig. 2), Δ_−+_ also represents the recent decadal change between the two epochs. We find that Δ_−+_(MPI) based on MPI(SST) is very weak, and therefore show only MPI(T_mix_) and the corresponding *ICI* in Fig. 3e,f. The Δ_−+_(WSH) decreases while Δ_−+_(VOR), Δ_−+_(*Z*_*26*_) and Δ_−+_(MPI) increase. According to the above cited works, these changes favor TC-intensification and contribute to increased Δ_−+_(*ICI*) (Fig. 3f), consistent with Fig. 2c that the intensification rates are positive during −PDO and negative during +PDO.

We evaluate the contributions of the individual variables to TC intensity changes. We first compare with the literature the magnitude of the contribution; we then quantitatively assess the contribution. The Δ_−+_(RH) ≈ +5% (Fig. 3a) is similar to the values previously reported as TC changes intensity^16, 19, 21, 22^; however, the magnitude is smaller than the values of ±10% that distinguish active and inactive TC periods^46^, and in SCS the climatological RH is high, ≈70% or more at 600 hPa, which is generally favorable for TCs irrespective of the phases of PDO. A sufficiently strong WSH > 10 m s^−1^ can rip a TC apart, weakening or even destroying it^23–28^. However, the Δ_−+_(WSH)≈−0.8 m s^−1^ (Fig. 3b) is small in magnitude relative to the climatological WSH of ~8 m s^−1^ over SCS^1, 47, 48^, and is weak compared to changes of 3~5 m s^−1^ found previously for significant changes in TC intensity^49^. The Δ_−+_(VOR) ≈ −2 × 10^–6^ s^−1^ (Fig. 3c) is similar to the values previously found in studies of TC intensity change^16, 50^, but the composite is insignificant in northern SCS. The increased Δ_−+_(*Z*_26_)≈ + 16 m (Fig. 3d) is a significant fraction (~25%) of the climatological mean *Z*_26 _= 50~60 m in northern SCS (Fig. S3). A shallow *Z*_*26*_ allows cold subsurface water to be more easily mixed and entrained to the surface by typhoon winds^41^ lowering the SST which in turn weakens the storm^1, 19, 29–33^.

**Figure 4:**
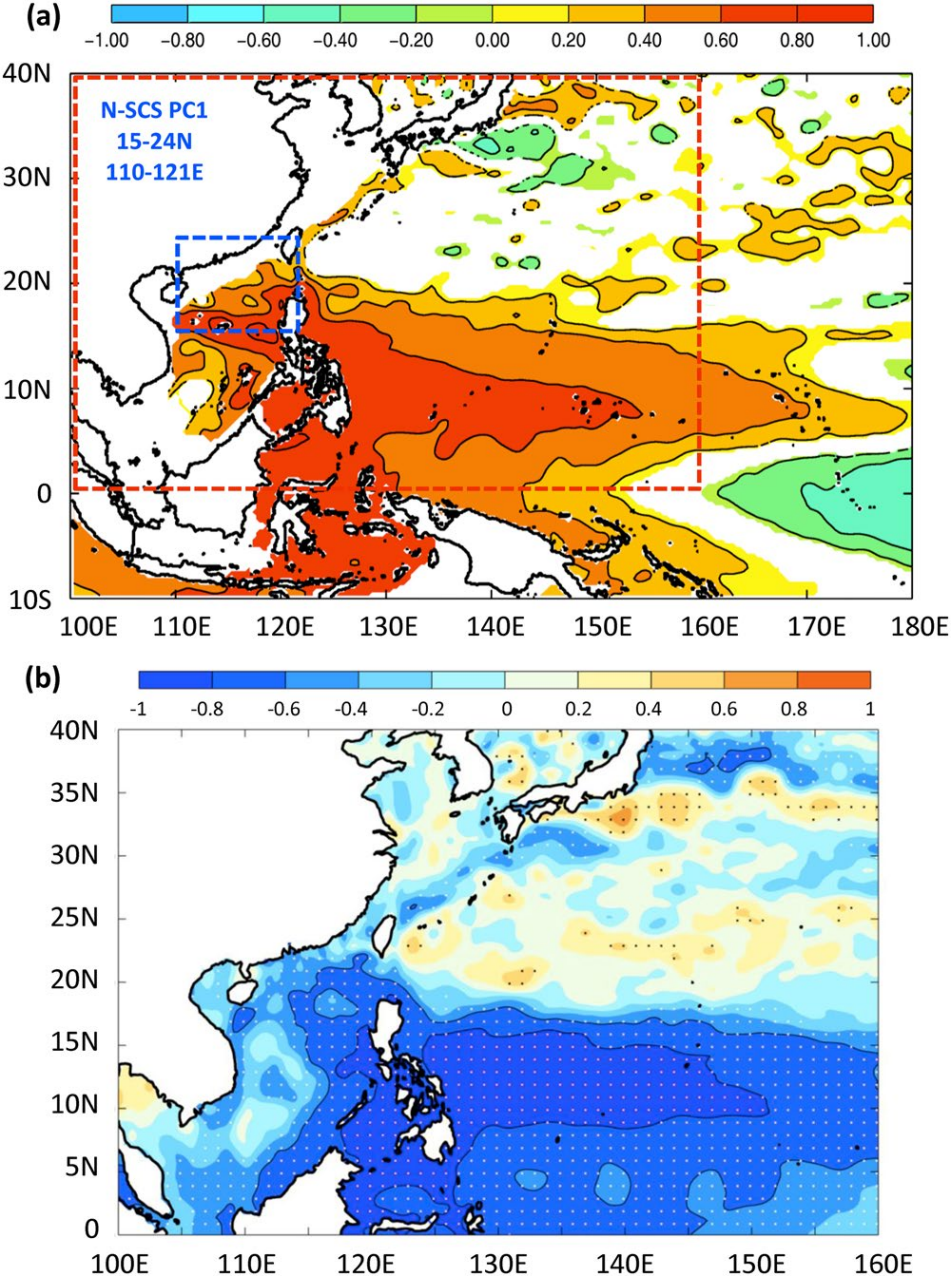
(**a**) Homogeneous correlation map of mode-1 northern SCS EOF(η’) (57%; domain shown in blue) with AVISO SLA in the western North Pacific from 1993–2013, showing that the SCS η’ is closely related to the SLA fluctuations over the warm pool, in particular to the fluctuations in the vicinity of the NECBF point near 12~13^o^N. (**b**) Correlation between NECBF with AVISO SLA in the red domain of (**a**); dots show correlations significant at the 95% confidence level. In SCS and warm pool, SLA drops (rises) as NECBF shifts north (south) [Chang and Oey 2012]. Maps plotted using MATLAB Version#R2012a (7.14.0.739) 64-bit (glnxa64) (https://www.mathworks.com/support/sysreq/previous_releases.html).

We extend the above composite analysis for a longer period before the satellite altimetry era i.e. before Oct/1992. To estimate η’ (hence *Z*′_26_), we note that SLAs in SCS and warm pool are correlated (Fig. 4a)^51^. In turn, both are closely related to the fluctuations of the North Equatorial Current bifurcation (NECBF) point near 12~13N east of the Philippines, such that sea level in SCS drops (rises) as the NECBF shifts poleward (equatorward)^51–53^, as shown in Fig. 4b. We therefore use a proxy of NECBF^54^ to project it to AVISO SLA and reconstruct η’ from 1982 to 2013. We limit our analysis to the period after 1980, since prior to that date the use of the NCEP data for TC parameters may be unreliable^55, 56^. We repeat the PDO-composite analysis. The results (Fig. S4) are similar to Fig. 3, showing that during −PDO (+PDO) years ICI is positive (negative) and the conditions are more (less) favorable for TC to intensify in SCS.

### Intensity changes

**Figure 5:**
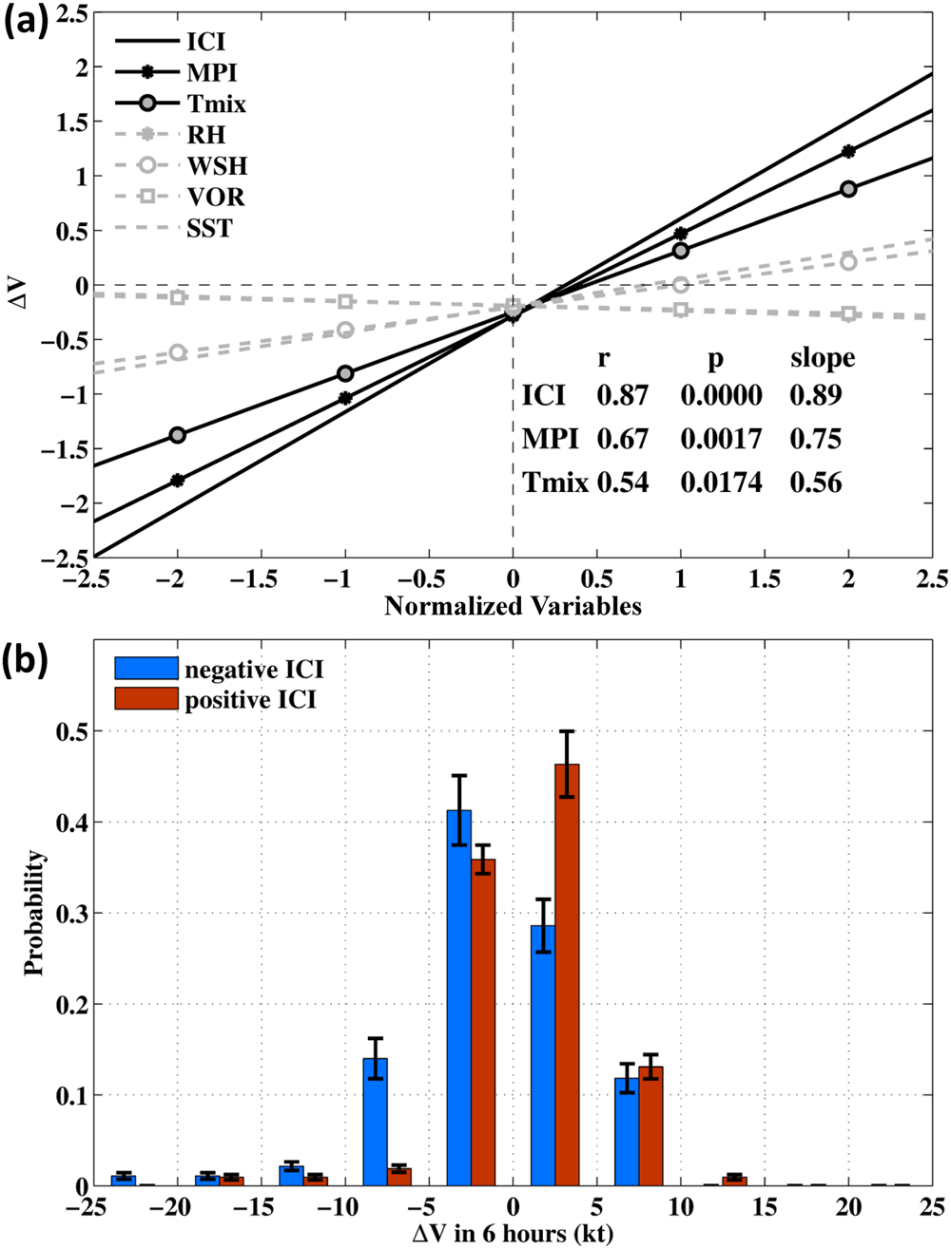
(**a**) Regression lines of normalized (by s.d. σ) 6-hourly intensity changes (ΔV) of LSTCs *vs*. the indicated variables, from 1993 to 2013. Legends show the *r*, *p* and slopes for *ICI*, MPI, T_mix_ which are all significant at the 98% confidence level: shown as solid dark lines; grey dashed lines are for insignificant regressions (*p* ≥ 0.53). (**b**) Probability distribution of ΔV sampled by positive and negative *ICI*s, from 1993 to 2013. Units are knots [1 knot (kt) = 0.51 m s^−1^] per 6 hours. Data are in bins of 5-kt resolution. Error bars show ±σ from the mean probability for that bin.

To quantitatively assess the influence of various environmental factors on TC intensity changes in SCS, we use the 6-hourly IBTrACS data to calculate the annual (typhoon season) mean intensification rates. We regress ΔV against the variables (Fig. 5a). We find that T_mix_ and MPI(T_mix_) are significant predictors of typhoon intensity changes in SCS. The SST, RH, WSH and VOR are poor predictors with large *p*-values. Except for VOR, their combined contribution to *ICI* is non-negligible however, and results in significant high correlation (*r* = 0.87, Fig. 5a); thus *ICI* accounts for 75% of the total variance of TC intensity change in SCS. These findings support the results of the composite analyses. Indeed, the probability distribution of intensification rates sampled by *ICI* (Fig. 5b) shows that the mean intensification rates between positive and negative *ICI*s are separated over a wide range up to |ΔV| ≤ 15 kt per 6 hours, and the mean intensification rates are positive for positive *ICI* and negative for negative *ICI*.

## Summary and Discussion

**Figure 6:**
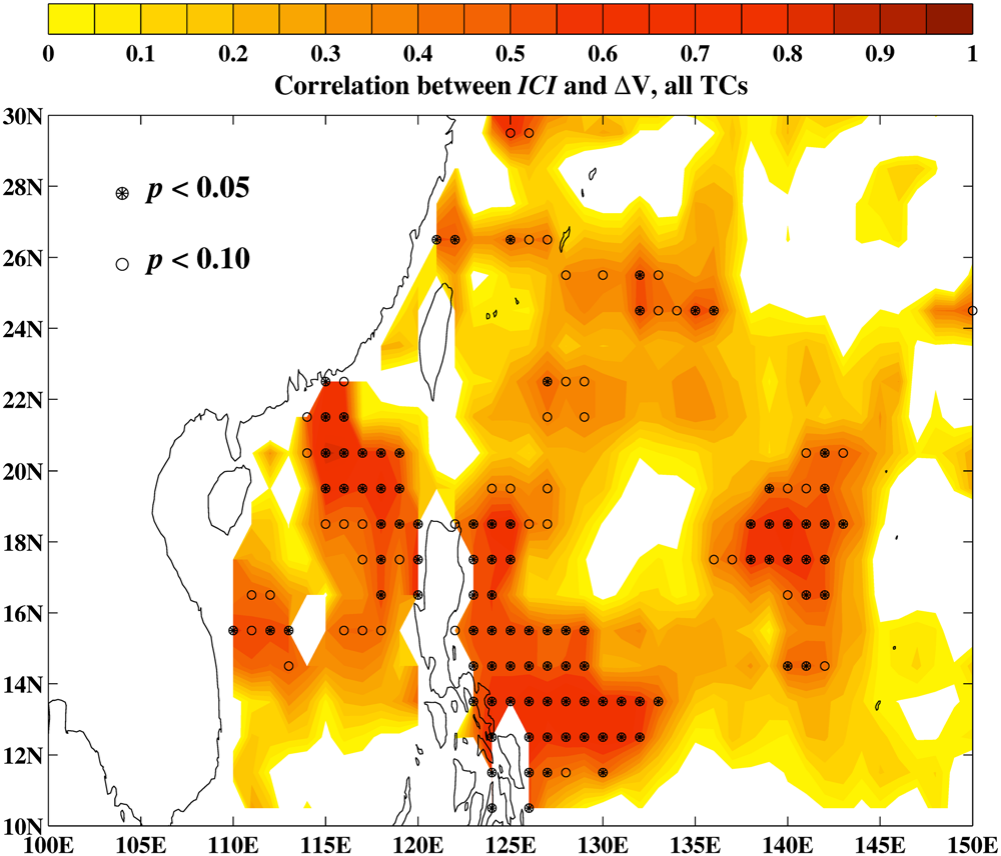
The correlation between *ICI* and ΔV. Filled (open) circles denote regions where the correlations are significant at the 95% (90%) confidence level. Maps plotted using MATLAB Version#R2012a (7.14.0.739) 64-bit (glnxa64) (https://www.mathworks.com/support/sysreq/previous_releases.html).

We have demonstrated that a greater percentage of typhoons entering the SCS (i.e. greater *R*_*ty*_) intensify during years of −PDO than +PDO. The dominant cause is a substantial (25%) deepening of the *Z*_*26*_ that tends to shelter the warm surface ocean from cool subsurface waters, which increases T_*mix*_. We noted before that T_*mix*_ increases with *both Z*_*26*_ and SST and Eq. (2) expresses the ‘sheltering’ property as described previously. Mechanistically, the deepened *Z*_*26*_ makes it more difficult for TC winds to mix and entrain cooler subsurface waters to the warm surface, increasing the MPI and allowing a greater percentage of TCs to intensify in SCS. Thus we develop *ICI* based on MPI(T_*mix*_) to describe the TC intensity changes. To assess *ICI* for regions other than SCS, we calculate the correlation between *ICI* and ΔV (Fig. 6) over the western North Pacific. It shows a highly significant band of correlation with *r* ≈ 0.6~0.8 from the Philippines Sea to northern SCS. Such a good predictability is clearly desirable for storm preparedness and risk analysis. As altimetry observations are now routinely available, the *ICI* can be used to formulate a more accurate TC-intensity prediction scheme.

Why does *Z*_*26*_ in SCS deepen for −PDO? Local changes may contribute. The *Z*_*26*_ can deepen due to a negative wind stress curl over SCS which depresses isotherms by Ekman downwelling. However, the surface wind stress curl (not shown) is positive during −PDO years in SCS and cannot contribute to the deepened *Z*_*26*_. The *Z*_*26*_ can also deepen due to heat transport into SCS from the western Pacific warm pool through the Luzon Strait. However, the Luzon Strait transport is *positively* correlated with PDO^52^, so that the heat transport deficit (surplus) during -POD (+PDO) years cannot cause *Z*_*26*_ to deepen (shoal). Instead, we argue that the deepening of *Z*_*26*_ in SCS is remotely driven (Fig. 4), closely tied to the deepening of the warm pool waters as PDO turns negative.

Sea level in the western tropical Pacific including the SCS has undergone a rapid rise in recent decades since the early 1990s^57^; the trend remains significant in SCS during the typhoon season (Fig.S5). The rise is *dynamically* caused by the strengthening of the easterly trade wind as part of the response to the recent global warming trend^6, 58^. Independent analysis^59^ also supports the idea that the strengthening of the Pacific Walker circulation since the mid-1970s can be explained by rising SST. Therefore, since as sea level rises *Z*_*26*_ deepens, both it and the rising SST lead to increased T_*mix*_, hence also a corresponding increase in *ICI*. Evidence that the *Z*_*26*_ has indeed increased more rapidly in recent decades compared to earlier decades can be seen by comparing the two *Z*_*26*_-composites (Figs 3d and S4d), suggesting a modulation in recent decades by the rapid sea-level rise previously identified^57^. Should negative PDO continue, and sea level and SST continue to rise, our study suggests that the SCS buffer may further weaken. In that case, one may expect potentially greater number of stronger landfalling typhoons along the SCS coastlines in the coming decades.

## Methods

Six-hourly typhoon locations and corresponding maximum wind speeds from 1951 to 2013 were obtained from the IBTrACS dataset^60^. Monthly atmospheric variables (for RH, WSH and VOR) were from NCEP/NCAR reanalysis data. Weekly satellite sea-level anomaly data from 1992/October to 2013 were downloaded from AVISO, and monthly averaged. Monthly World Ocean Atlas (WOA) data is used to estimate climatological *Z*_*26*_. The SST from GHRSST (1982–2013, 1/4^o^ × 1/4^o^) is used in the calculations of various metrics related to TC-intensity. Monthly PDO time series^8^ is used to compute positive and negative PDO composites (i.e. arithmetic averages) of various quantities. We focus on long-term variability and, except for the IBTrACS data, monthly climatology is removed from each time series. All quoted trends and correlations are given at the 95% confidence level i.e. the probability that the trend or correlation is due to randomness of the time series is *p* < 0.05.

We develop an index to describe the environmental effects on TC intensity change:

$$ICI=F({G}_{\varpi };{G}_{RH};{G}_{MPI};{G}_{WSH}),$$

where *F* and the *G*’s are some functions of the subscripted variables, ϖ = absolute vorticity at 850 hPa (s^−1^), *RH* = relative humidity at 600 hPa, *MPI* = MPI(T_mix_), and WSH = |**V**_s_|. The functional *F* is a product of *G*’s, and the *G*’s some fractional power functions, i.e. ratio of polynomials of the variables raised to some power. Using the 6-hourly IBTrACS data, the exponents are calculated using an iterative Newton algorithm to maximize the percentage variance of ΔV described by *ICI*, yielding Eq. (3). The effect of ${G}_{\varpi }$ is very weak and is omitted in (3). We also considered including the effect of the TC translation speed on TC intensity change^28^; it too has only minor effects. We compare *ICI* with GPI. In northern SCS, *ICI* accounts for 75% of the total variance of ΔV, compared to 30% for GPI.

## Electronic supplementary material

Supporting Information
