# Supplementary material for: Sea level rise, surface warming, and the weakened buffering ability of South China Sea to strong typhoons in recent decades
Source: Sci Rep. 2017 Aug 7;7:7418. doi: 10.1038/s41598-017-07572-3 (PMC5547110; doi:10.1038/s41598-017-07572-3)
Supplement: Supplementary file 1 — Supporting Information [file 41598_2017_7572_MOESM1_ESM.pdf]

Supporting Information for

Sea level rise, surface warming, and the weakened buffering ability of South China Sea to strong typhoons in recent decades

Jingru Sun[1], Leo Oey[2,3,*], F-H Xu[1] & Y-C Lin[2]
1: Tsinghua University; 2: National Central University; 3: Princeton University
*Corresponding Author: lyooey@gmail.com

Introduction

This supporting information file includes text and figures which support the results described in the main text.

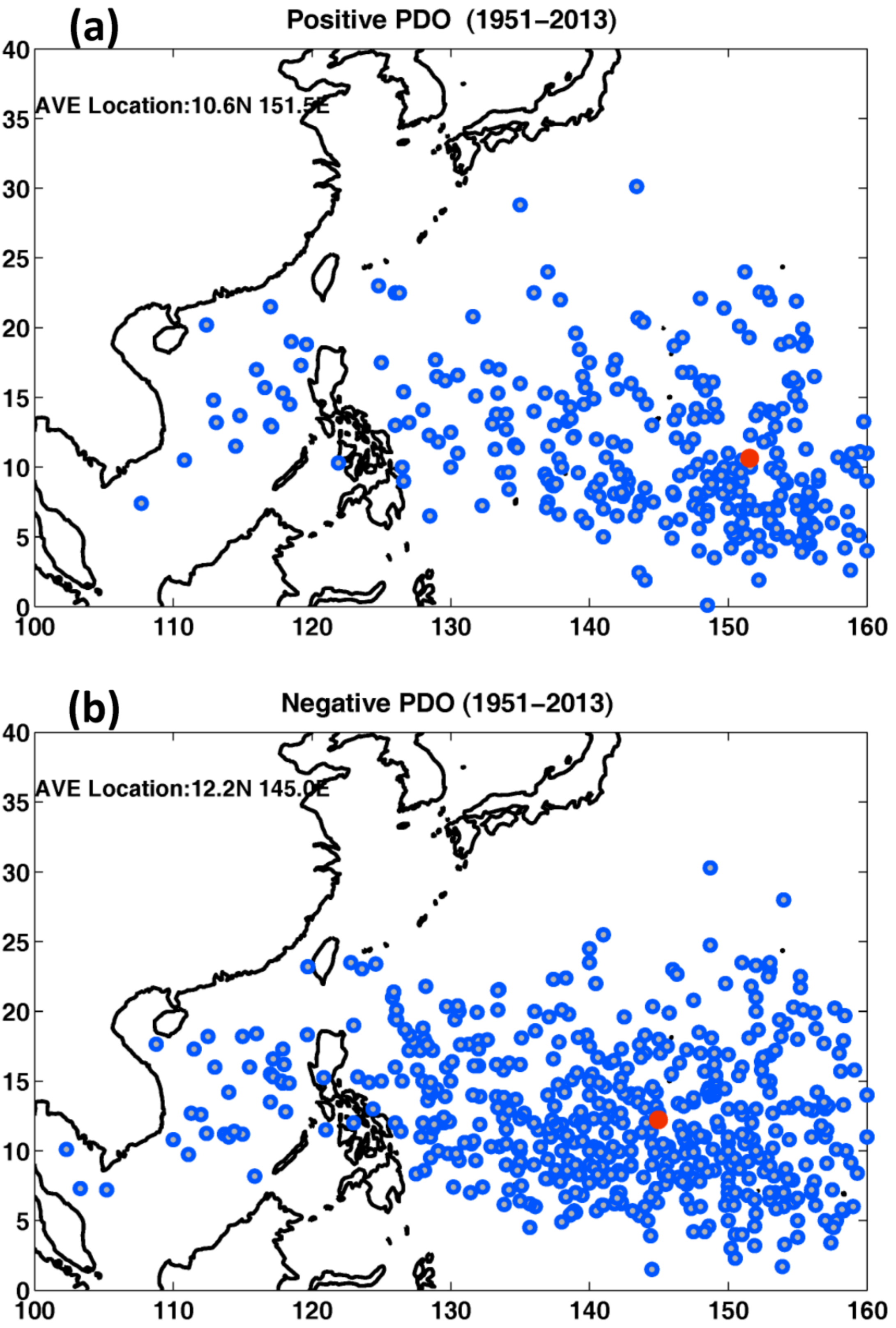

Fig.S1 Typhoon-genesis locations grouped in (a) +PDO and (b) -PDO years. During -PDO years, typhoons tend to form farther west in the North Pacific, and the mean cyclogenesis location is at 145°E, 12.2^{o}N, while the mean location is shifted east-southeastward to 151.5^{o}E, 10.6^{o}N during +PDO years: there is a shift from -PDO to +PDO of 6.5^{o} eastward and 1.6^{o} southward. Maps plotted using MATLAB Version#R2012a (7.14.0.739) 64-bit (glnxa64). (https://www.mathworks.com/support/sysreq/previous_releases.html).

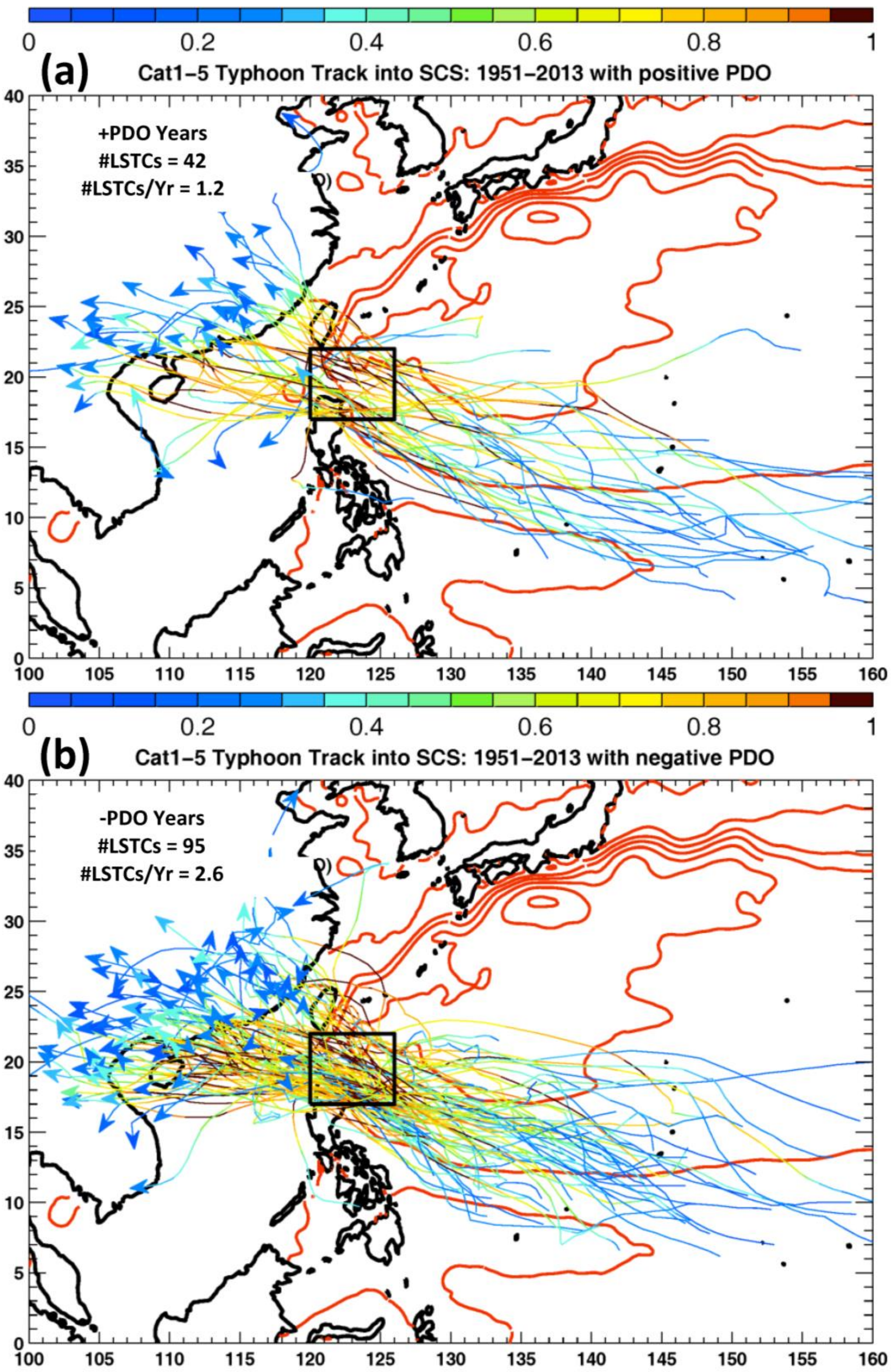

Fig.S2 Tracks from IBTrACS observations of LSTCs (see Fig.1a) grouped into (a) +PDO and (b) –PDO years for the period 1951-2013. For each track, colors show 6-hourly wind speeds normalized by the maximum wind speed for that track; darkest brown color corresponds to normalized wind speeds > 0.95, and indicates where and when the typhoon was near its LMI. Red contours show mean absolute dynamic topography MADT (*m*) from AVISO http://www.aviso.oceanobs.com/. Maps plotted using MATLAB Version#R2012a (7.14.0.739) 64-bit (glnxa64). (https://www.mathworks.com/support/sysreq/previous_releases.html).

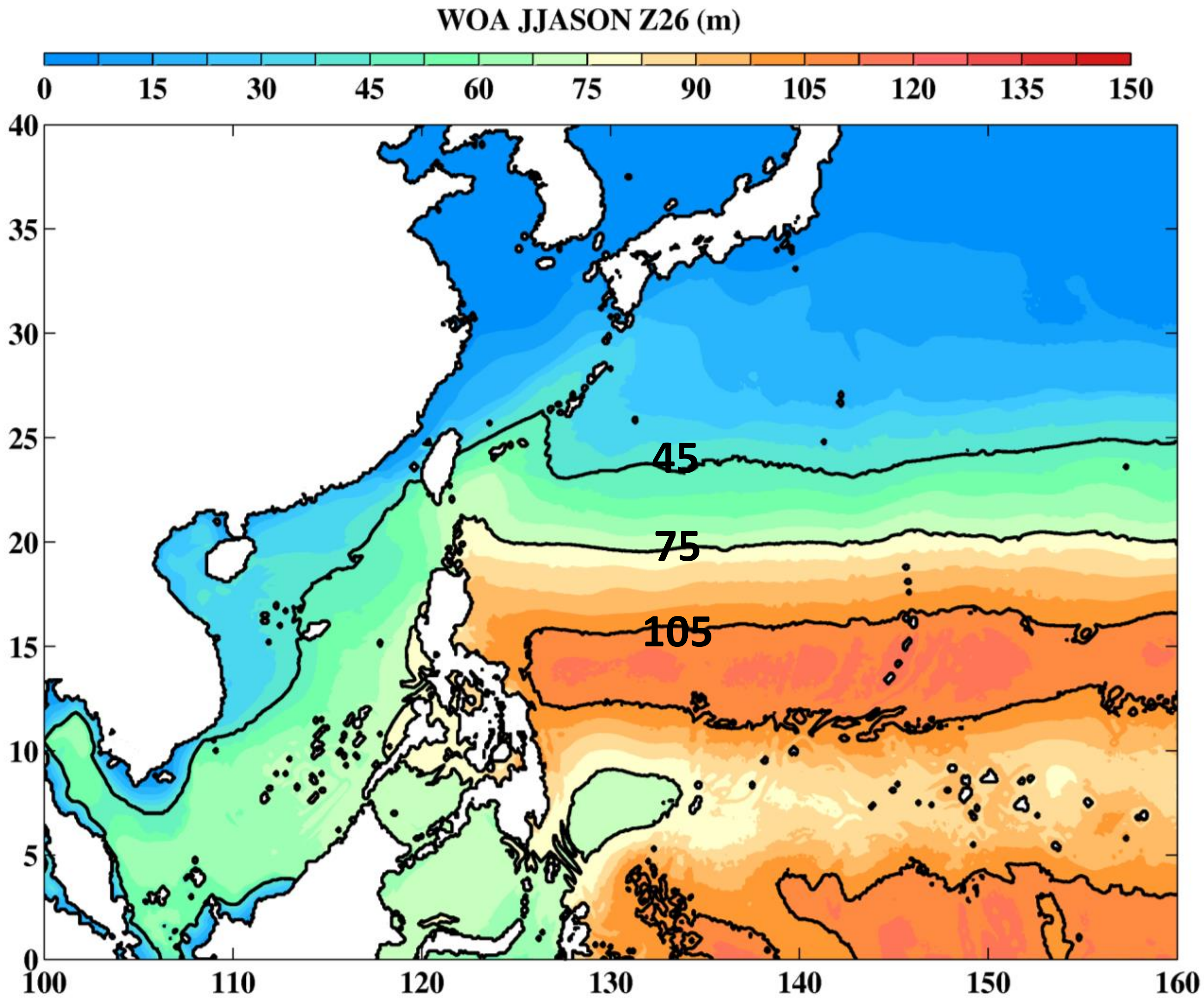

Fig.S3 Climatological (JJASON) mean Z_{26} (m) from WOA. Maps plotted using MATLAB Version#R2012a (7.14.0.739) 64-bit (glnxa64).
(https://www.mathworks.com/support/sysreq/previous_releases.html).

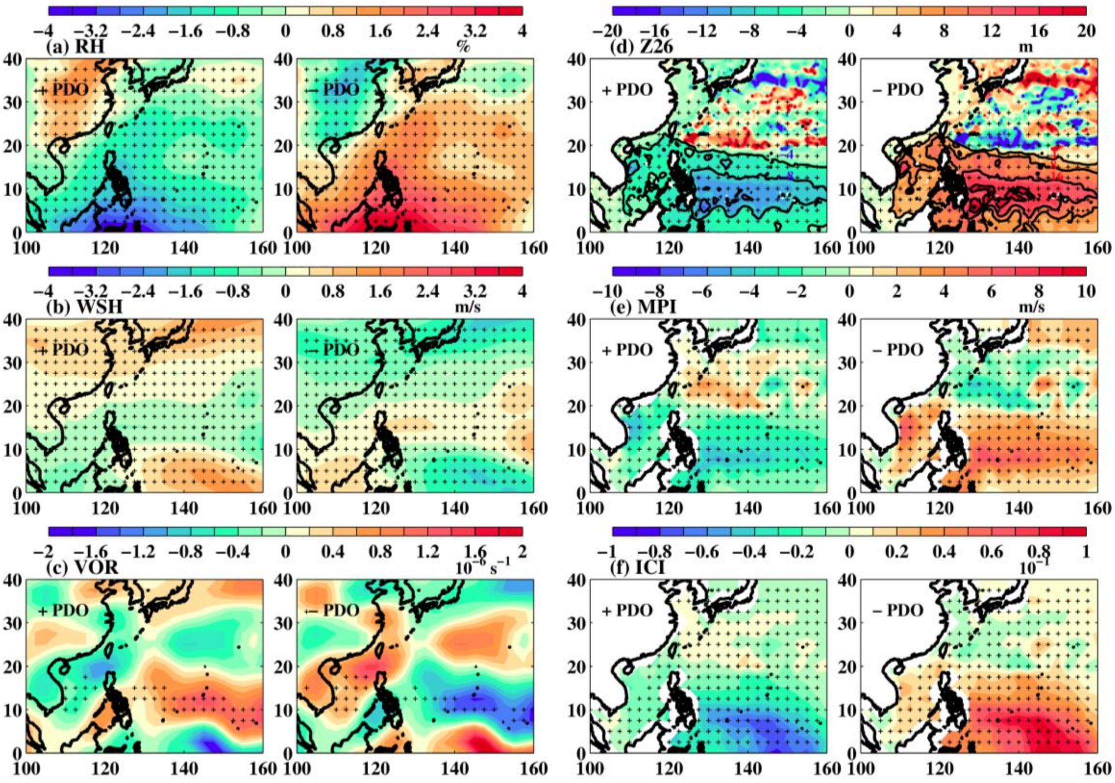

Fig.S4 Composites of (a) RH, (b) WSH, (c) VOR, (d) Z_{26}, (e) MPI and (f) ICI for +PDO and –PDO from 1982 to 2013 using the reconstructed η' (hence Z_{26}') (see text). These should be compared with the 1992-2013 composites using the full AVISO SSHA (Fig.3). Note the different scales used in (d) and Fig.3d. The reconstructed Z_{26}' is 30~50% weaker than Fig.3d; however, its contribution to ICI is still dominant. Maps plotted using MATLAB Version#R2012a (7.14.0.739) 64-bit (glnxa64). (https://www.mathworks.com/support/sysreq/previous_releases.html).

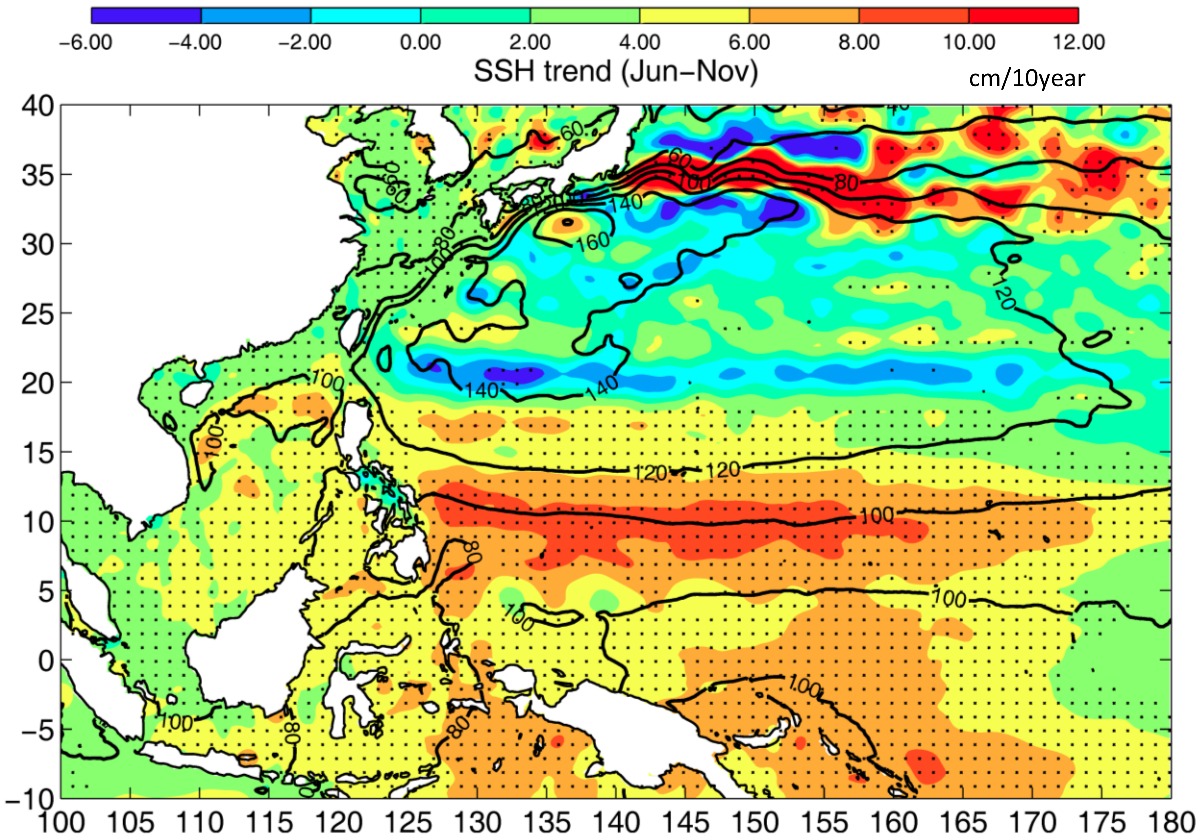

Fig.S5 Sea-level trend from AVISO from 1993-2013 (color shading). Dots show trends at the 95% confidence level. Contours are the long-term mean of Absolute Dynamic Topography (cm) from AVISO. Maps plotted using MATLAB Version#R2012a (7.14.0.739) 64-bit (glnxa64). (https://www.mathworks.com/support/sysreq/previous_releases.html).
